# Forty years of research concerning children and youth in Greenland: a mapping review

**DOI:** 10.1080/22423982.2017.1323526

**Published:** 2017-06-29

**Authors:** Mia Glendøs, Peter Berliner

**Affiliations:** ^a^ Department of educational psychology, Aarhus University, Copenhagen, Denmark

**Keywords:** Systematic review, research methods, knowledge conduction, children and youth, mental health in Greenland, psychology related issues

## Abstract

**Objective**: This study undertakes a mapping review of mainly concerning children and youth in Greenland in the period 1976–2016 and reflects on how the research has been conducted and knowledge thereby created about children and youth in Greenland, as well as how the research has been developed over time.

**Design**: 16 online databases; five journals; publication lists originating from seven organisations and ten prominent researchers; and local network and references were used in the search and subsequently screened through the scoping criteria. 342 publications were included, encompassing knowledge based on empirical research on children and youth in Greenland within the broader field of psychology.

**Results**: The majority of studies, 71%, were conducted through quantitative methods. The qualitative research is represented in 22% of the studies and participatory and action-orientated research is represented in 7% of the studies. The most prominent themes in research concerning children and youth in Greenland were physical problems, which were found in 38% of the studies.

**Conclusions**: The result reflects a consistent objectivity and quantitative methodology in health research in Greenland since 1991.The health research thus represents a united research community with a shared methodological research approach, while the local participatory action research projects all appear differentiated. While health research covers a spectrum of psychology related objectives, the methodology traditions reveal a specific kind of knowledge, which has come to determine how the mental health of the Greenlandic children is perceived. We believe that more qualitative and locally grounded approaches need to be organised in order to produce a more nuanced knowledge of the Greenlandic children and youth.

Research has been produced for centuries in order to gain knowledge about indigenous people in the Arctic region. The scientific fields tend to overlap and both social and biomedical health research involve topics related to the field of psychology, such as mental well-being, societal transformational life circumstances, suicide, psychopathology, etc. Former literary reviews (a total of 22 were found) have been concerned with studies in specific areas, mostly health or biomedical research, and missing in the reviews is a gathering of mental, social, societal and biological health research.

The 22 former reviews concerning children and youth in Greenland cover the following topic areas: causal protective factors contributing to indigenous mental health [1], diet [2–4], physical place of birth [5,6], economic and political issues [7,8], suicide knowledge [9,10], the health research in indigenous people in the whole circumpolar area [11,12], identification of indigenous peoples [13], placement of children in care [14], catalogues of health research in Greenland [15], medical bibliography [16], studies of diseases and sickness [17,18], children’s ill-health disease patterns [19,20], health, sickness and behaviour of socially vulnerable groups [21], and environmental coherence with the epidemiological literature [22]. This present review samples 342 studies concerning children and youth research in Greenland published in the forty-year period from 1976 to 2016, all associated with psychology related issues including mental health, health, social and biomedical studies.

In this article, we map out the existing empirical knowledge from the 342 studies, demonstrate our applied rigor-systematic search process and screening strategy, and present the result in order to answer the main review question: “What psychology related research exists on children and youth in Greenland between 1976 and 2016 and how does the research generally develop through this time period?” As the term *psychology* is not easily distinguished from other academic disciplines, we also included studies in the fields of health care, demography and related social science areas. The result will be presented and discussed using the following two interrelated focus questions:

- Which empirical methods are used?
- Which themes are represented?

This article will answer the main research question and the two focus questions. Firstly, we will describe the scope of the review. Secondly, we will elucidate the search process and the screening procedure. Thirdly, we will map out the results related to the focus questions. Fourthly, and finally, we will reflect on how knowledge about children and youth in Greenland is created in research and developed over time.

## Scope

The scope of the review study was determined through choices made regarding the research areas associated with the field of psychology, the interest in children and youth, the 40-year period and the location and linguistic universe.

### Research areas associated with the field of psychology

Psychological health is not an isolated factor in human life but closely interrelated to social and physical issues. This review thus includes a variety of methodological research traditions, all related to psychological research concerning children and youth in Greenland. Among these are studies of statistical demographics, socioeconomics, pathology, behaviour, public health, culture, linguistics, psychiatry, family, education, mental health, medical health and community.

### The interest in children and youth

We consider the age limits within the category of children and youth to be from newborn to 25 years old. Additionally, we chose to include studies that also consider adults and social structures in children and young people’s lives, because we consider the individual to be interrelated with their social surroundings. The health and lifestyle of the grown-ups in society and the adults in the institutions of school and day care service are of significant importance to the lives of the children and youth. Furthermore, many studies involved a mix of adults, young people and children, and distinguishing the answers of children and youth in these studies was impossible. Therefore, studies that did not focus directly on children and youth were also included in the review if their objectives influenced the lives of children and youth; for example, studies on the teachers’ educational system or on the well-being among adults.

### The 40-year period

The review includes literature from January 1976 to September 2016. This time span was chosen both as a consequence of specific available data and for its relevance to related historical events. The historical events determining the period of the study relate to the onset of Home Rule in 1979. We expected that political debate and the growing public support for greater Greenlandic independence in the years before the new Home Rule would correlate with published research on the lives of children and youth in Greenland.

### Location and linguistic universe

There have been several instances of research collaboration between Canada and Greenland; however, our scope was to focus exclusively on research that included Greenland.

The review contains texts written in Danish, Swedish, Norwegian, and English. Texts in the Greenlandic language – Kalaallisut – are reviewed in their Danish or English versions. We do not know of any research on children or young people that has been published only in Kalaallisut.

## Data collection

The search was undertaken in two stages: a main search and an additional search. The main search was conducted through global databases, psychology journals and journals concerning Greenland or the circumpolar region. The additional search provided further potential references (SPR) through searches within relevant organisations, selected researchers’ publications lists and a contributing snowball approach via reference lists from included texts, networks and emerging associated literature included in library databases.

### The main search

The main search included the following 16 databases in order to obtain the broard research field of the scope: CBCA (*Canadian Studies, Education*), ERIC (*Education Resources Information Center*), DAAI (*Design and Applied Arts Index*), ebrary/ebooks, IBSS (*International Bibliography of the Social Sciences*), LLBA (*Linguistics and Language Behavior Abstracts*), MEDLINE®, PILOTS (*Published International Literature on Traumatic Stress*‎), ProQuest (including: ProQuest Dissertations & Theses Global /Education Journals /Research Library /Science Journals), PsycARTICLES, PsycINFO, Social Services Abstracts, Sociological Abstracts, and Worldwide Political Science Abstracts. The search process in these databases was repeated four times with slightly expanded keywords over a three-year period from October 2014 to May 2016. The year and command lines for each search are presented below:

2014: ((child* OR schoolchild* OR pupil* OR adolescen* OR youth* OR famil*) *AND Greenland AND* (health* OR psycho* OR social*) *AND* (vulnerable* OR exposed* OR “at risk*” OR abuse* OR neglect* OR suicide* OR resili*)) *AND peer*(*yes*) *AND pd*(*>19531231*) = 111 hits.

2015: (barn* or børn* or skolebarn* or skolebørn* or elev* or teenager* or ung*) *and Grønland and* (sundhed* or psyko* or social* or syg*) *and* (udsat* or sårbar* or risiko* or mønsterbr* or misbr* or neglect* or selvmord* or robust* or resili*) = 197 hits.

2016: ((child* OR “schoolchild*” OR pupil* OR adolescen* OR youth* OR famil*) *AND Greenland AND* (health* OR psycho* OR social*) *AND* (vulnerable* OR exposed* OR “at risk*” OR abuse* OR neglect* OR suicide* OR resili*)) *AND peer*(*yes*) *AND pd*(*>19531231*) *OR* (barn* OR børn* OR skolebarn* OR skolebørn* OR elev* OR teenager* OR ung*) *AND Grønland AND* (sundhed* OR psyko* OR social* OR syg*) *AND* (udsat* OR sårbar* OR risiko* OR mønsterbr* OR misbr* OR neglect* OR selvmord* OR robust* OR resili*) = 348 hits.

2016: *ab*(Greenland* OR greenland* OR Kalaallit Nunaat OR East* Greenland* OR Østgrønland* OR Inuit*) *AND ab*((teacher* OR educa* OR pedagog* OR pædagog* OR lærer*) *OR* (child* OR “schoolchild*” OR pupil* OR adolescent* OR youth* OR family*) *OR* (barn* OR børn* OR skolebarn* OR skolebørn* OR elev* OR teenager* OR ung*)) *AND ft*((health* OR psycho* OR social*) *OR* (sundhed* OR psyk* OR social* OR syg*) *OR* (vulnerable* OR exposed* OR “at risk*” OR abuse* OR neglect* OR suicide* OR resili OR strong*) *OR* (udsat* OR sårbar* OR risiko* OR mønsterbr* OR miser* OR neglect* OR selvmord* OR robust* OR resili OR stærk)) *AND pd*(*>19760131*) = 615 hits.

The four searches in the databases produced 1,271 hits in total, of which many were duplicates and many others could be excluded when the title clearly indicated that they did not concern children and youth in Greenland. As many published research articles on Greenland are not included in the major databases [23], five journals was selected, based on our knowledge of publications in the field, to complement the main search: *Arctic* (from AINA, Arctic Institute of North America), *CSRG* (Cultural and Social Research in Greenland), *IJCH* (International Journal of Circumpolar Health), *INUSUUK* (the Arctic Research Journal of the Department of Education, Culture, Science and Church in Naalakkersuisut, Greenland Self-Government), and the Danish psychology journal *Psyke & Logos*. Unlike the database searches, which used a command line with multiple keywords, the search in the chosen journals could proceed only by using a single keyword for each search. For three of the journals, *Arctic, IJCH* and *Psyke & Logos*, the keyword “Greenland” was used, also in translated versions of Greenlandic, Danish, Swedish and Norwegian. In *CSRG* and *INUSUUK*, all published articles were screened by title before their inclusion in the sample of references.

### The additional search

Seven organisations in Greenland and Denmark that conduct Greenlandic research were included in the additional search. These were: Ilisimatusarfik (the University of Greenland), MIO (National Advocacy Center working for Children’s Rights), MIPI (the Documentation Center on Children and Youth), PAARISA (Governmental department of Health and Prevention), KVUG (the Commission of Scientific Research in Greenland, under the Ministry of Higher Education and Science), SFI (the Danish National Center for Social Research) and NIPH (the National Institute of Public Health at the University of Southern Denmark). The search in the publication lists of these organisations was primarily conducted using a single keyword in each search, and several searches were conducted using the words *Greenland, children* and *youth* (also searches with the same words translated into Danish).

We included a search in the publication lists of 14 selected researchers on public and mental health in Greenland. The 14 researchers, basically selected for their productivity in the field, were:

- Birgit Kleist Pedersen, Department of Culture, Language and History, University of Greenland, Ilisimatusarfik
- Birgit Niclasen, Government of Greenland principal investigator of health behaviour in school-aged children
- Christina Warrer Schnohr, Department of Public Health, University of Copenhagen
- Elizabeth Rink, Department of Health and Human Development Montana State University
- Else Christensen, Danish National Centre for Social Research (SFI)
- Gitte Adler Reimer (Tróndheim), Department of Cultural and Social History, University of Greenland, Ilisimatusarfik
- Inge Lynge, Dronning Ingrid’s Hospital, Nuuk
- Inger Dahl-Petersen, National Institute of Public Health, University of Southern Denmark
- Karla Jessen Williamson, Arctic Institute of North America (AINA) and University of Saskatchewan, Saskatoon, Canada
- MarieKathrine Poppel, Department of Social Work, University of Greenland, Ilisimatusarfik
- Peter Berliner. Departments: Educational Psychology at Aarhus University and Social Work at University of Greenland, Ilisimatusarfik
- Peter Bjerregaard, National Institute of Public Health, University of Southern Denmark
- Ruth Montgomery-Andersen, Department of Culture & Social History, University of Greenland, Ilisimatusarfik
- Tine Curtis, Centre for Prevention in Practise, Aalborg, and Aalborg University.

Based on titles and abstracts, 62 new potential references were included from this search. Conference papers were not included because these seldom had a form matching the other studies.

Through snowball search supplementing the additional search, 73 potential references were found. Here, two approaches were used: a Library search and a Network search. The Library search was conducted via the systematic search when reference lists in the included studies based the search in the Aarhus University Library database. The Network search approach was conducted via another Greenlandic research project in which colleagues, project supervisors, article reviewers and professional social workers contributed with relevant references on other research carried out in Greenland.

## Screening procedure

Two levels of screening procedures were used in the process, as represented in the boxes to the right of the flow chart model. At the first level, the process was conducted via a titles and abstracts screening. At the second level, the process included introduction, summary or full-text screening if the themes, research methods or results were unclear. The studies all had to meet the three screening criteria for inclusion:

1. The study must present knowledge on children and youth in Greenland.
2. The study must be within a broad field of psychology, including overlapping scientific fields.
3. The study must be based on empirical research.

The first criterion underlines the presented scope. Texts included that did not directly concern children and youth must involve studies considering family issues of social, economic and health relations; studies on institutional matters concerning day care or educational institution; and studies on a national, municipal or community level relevant to the living conditions of children and youth.

The second criterion also underlines the presented scope by defining the field of psychology as overlapping with other scientific traditions. Natural science, arts and social science can all form part of the field of psychology [24].

The third criterion was defined through careful consideration of the methodological quality of the empirical research foundation of the texts. Thus, empirically founded research must be transparent, reliable and coherent. The present review includes empirically founded articles, books, book sections, PhD dissertations and government reports on a peer-reviewed scholarly or semi-scholarly level.

**Figure 1.**
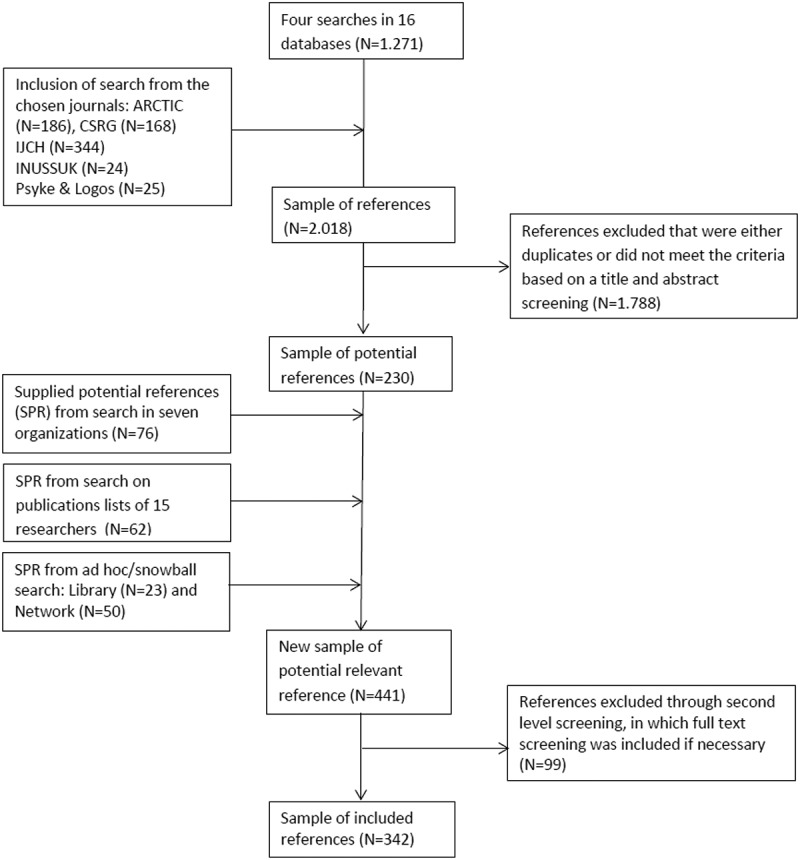
Flow chart for the search location and selection of relevant studies.

The included 342 studies did not reflect 342 research projects since huge research projects such as national population surveys and comprehensive community action research generate more than one study. Nevertheless, each study concentrates on different elements of the main research project. The flow chart model below illustrates the systematic process of inclusion and exclusion of references:

The boxes in the central column of the flow chart model in Figure 1 represent how the number of references was developed beginning at 1,271 references from the database searches to the included review sample of 342 references. The left column boxes illustrate the search locations of both the main and the additional search, and the right column boxes illustrate the process of exclusion. The exclusion process was conducted through a screening procedure with criteria that will be explained later. First, the main and additional search processes will be explicated.

## Systematic mapping through the research focus questions

The sample of 342 studies was analysed focusing on the main review question of defining the existing psychology related research on children and youth in Greenland between 1976 and 2016. The method used for this mapping analysis was as follows: in an Excel program, each study was listed according to publication year, author(s), title, focus, method, result and main conclusions. After this, colons were inserted to identify the frequencies, similarities and diversities in the methods and research themes, addressing the two subsidiary research questions to define what kind of data inquiry method was generally used, and what themes were represented in the studies.

### Data inquiry methods

The data inquiry methods are related to the methodologies and theoretical traditions based on general epistemological approaches and the ontology of the researcher. Thus, differing approaches in method, methodology and theory create different representations of the particular field of research. We have synthesised the studies in five categories which will be thoroughly elaborated. First, we will illustrate the frequency of each category of inquiry method in a circle diagram.

**Figure 2.**
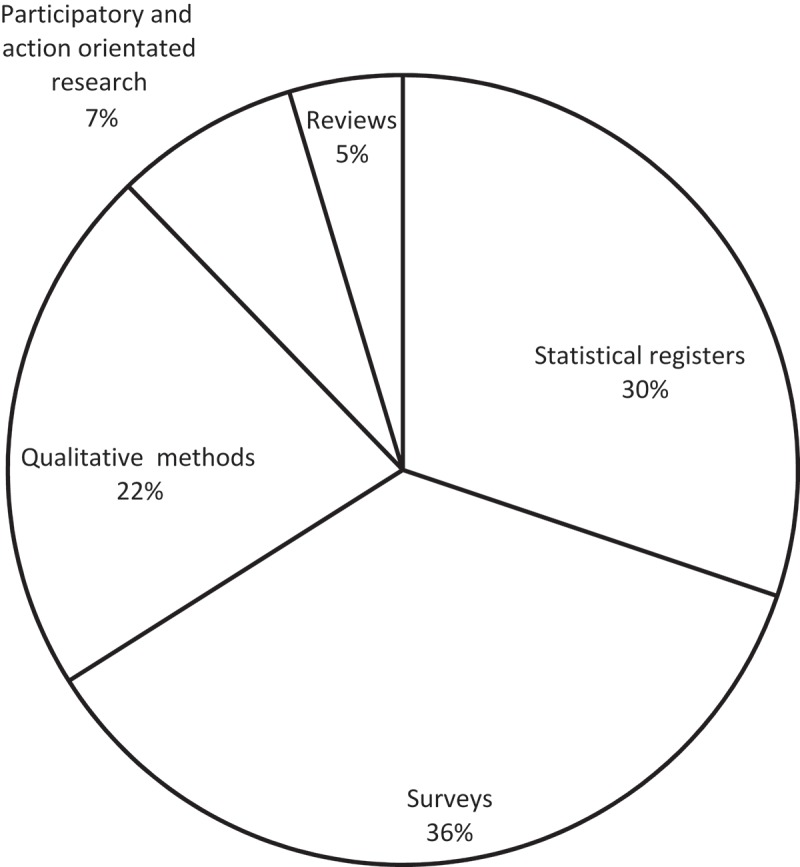
Frequency of inquiry methods in psychology associated research concerning children and youth in Greenland 1976–2016.

The five categories presented in Figure 2 illustrate the most frequently applied research methods in the studies. Many studies operate with more than one data inquiry approach, and 14% of the studies use a mix of inquiry approaches from both quantitative and qualitative research methods. Roughly dividing the research between quantitative and qualitative approaches, we find that a significant majority of 71% of the inquiry methods in the studies is based on a quantitative methodology, related through the methods of statistical registers, surveys and other reviews. The distinction between quantitative and qualitative research often appears as a rigid dichotomy [25], and their distinct definition often varies according to differing research approaches. We believe that each approach needs to be appreciated on its own terms for its strengths and limitations, and for the knowledge to which it contributes. Traditionally, natural and health sciences use quantitative inquiry methods with surveys and statistical registered analysis, whereas the social sciences and anthropology use a more qualitative approach. The dominance of quantitative research approaches thus reflects a majority of the studies found in health sciences.

The category called *Statistical registers* in the circle diagram in Figure 2 represents research based on national or organisational registers. This can be from Statistics Greenland’s free databank which carries statistics and information about Greenlandic society, the Greenland police annual report containing records of reported crimes and use of police resources, hospital records of stored medical tests and registers of births and deaths, as well as municipal and school statistical reports.

The category called *Surveys* encompass many quantitatively defined methods including self-reporting questionnaires, questionnaire-based interviews, structured telephone interviews, cross-sectional studies, and some cohort studies using questionnaires, anthropometric measurements (height, weight, waist and hip circumferences), and samples of biological specimens (blood, urine, and fat biopsies). The objective of surveys is to gather information on physical measures and self-rated health, behaviours, knowledge and attitudes [25]. Answers are carefully recorded from a large number of people who have been asked the same questions or have participated in the same tests, and the participants are often selected through random sampling techniques.

The category called *Qualitative methods* includes the empirical material of historical analysis, case studies, open and semi-structured interviews, life-story interviews, field studies, archaeological studies, microbiological analysis, studies of art, observations, focus-group interviews, and cohort studies using less structured interview techniques. Qualitative inquiry is useful in producing authentic [25], situated [26], and specific knowledge about individuals, relations and smaller communities.

The category called *Participatory and action orientated research* concerns research with an interventional agenda. Methods or methodologies covering various change orientated research approaches all have in common that researchers and practitioners ideally collaborate to initiate change in practise and produce knowledge about practise [27]. The participatory and action orientated research in the context of Greenland is often connected with a community focus founded in local utilisation. Researchers have a responsibility to carry out research that is meaningful, socially responsible and committed to a set of disciplined, material practices that produce radical, democratising transformations in the civil sphere. These practices involve collaborative dialogue, participatory decision-making, inclusive democratic deliberation, participation and representation of all relevant community members [28].

### Themes represented in the studies

In the 342 included studies of empirical research on children and youth in Greenland, 126 singularly included children and youth. The rest of the studies encompassed family studies and population studies which included a mix of children, youth and adults, or studies that focused more broadly on the well-being of adults or on societal structures, school systems, institutions and other conditions influencing the lives of children and youth.

The 342 included studies represent a variety of research themes; however, some themes are more frequently represented than others. The review analysis was conducted by coding the focus and aim of each study as stated in its title, abstract, introduction or summary. The codes were then sorted into categories (which we describe as themes) according to similarities. Thus, the themes present a general outline of the central topics in the research. A total of 17 themes were constructed from the codes of each study focus. Themes approached differently in the specific studies are therefore in this analysis clustered together. The themes and their representation frequencies are illustrated in the next diagram.

**Figure 3.**
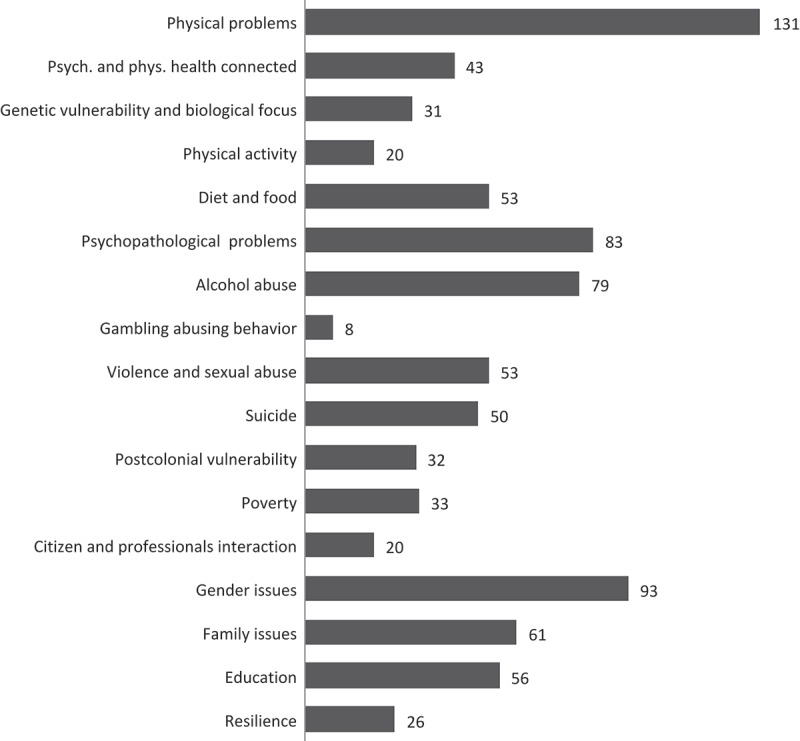
Themes in the studies on children and youth in Greenland, 1976–2016.

Figure 3 illustrates the themes represented in the N=342 sampled studies presenting psychology related empirical research on children and youth in Greenland in the 1976–2016 period. Several themes can be included in one study, and some themes are determined as a primary and some as a secondary focus in each study. We will explore each theme in the following section.

#### Physiology related themes

The theme of *Physical problems* covers research on physical damage, vulnerability, disease, sickness and medical care. This theme is the most frequently represented in the studies, with 131 hits. This over-representation reflects the many public health studies. Since Nuna Med ’91, the first conference on medical health research in Greenland, medical health research networks have been well established compared to other fields of research in Greenland. Professor Peter Bjerregaard characterises [23] this field as having a few permanent residents and numerous guests, all using similar methodologies (ibid., p.131). In 1997, the Greenland Center for Health Research was established [29], and in 2004 a declaration on priorities in health research policy pointed out what future public health research should include: the influence of social development on public health, children in Greenland and their healthcare behaviour, social and geographical inequality, prevention and intervention research, and human biology research [23]. The 2004 declaration acknowledges the relationship between physical and mental health, and the theme of Figure 3, *Psychology and physiology related health* includes 43 studies, all noticing the interdependent relationship between mental and physical health conditions.

Research on themes of *Genetic vulnerability and biological focus* is clustered in a united theme of 31 studies, while 20 studies include a focus on *Physical activities*. With the exception of a community project in 2001 called MATU [30], in which young male offenders were trained as sports athletes and subsequently transformed their community image and popularity, most of the studies on physical activities focused on measuring levels of physical activity in everyday life, e.g. through survey questions like “how much do you exercise every day?”

53 studies include a focus on *Diet and food*. The majority of the research methods used were quantitative, including review, survey and register statistics. Seven studies in the theme used qualitative methods, one used exclusively quantitative methods [31] and the other six combined quantitative and qualitative approaches [32–37]. The primary way this theme is inquired may thus miss out the psychology related issues regarding diet and food, like traditions, habits, season and location related possibilities, self-esteem, social relations and comfort, which could be revealed more detailed and context related in qualitative method approaches.

#### Themes related to mental distress

83 studies focus on *Psychopathological problems*. 66 of these relate to vulnerability in psychiatric or psychopathologic development, while 17 relate to actual psychopathological disorders. 27 of the studies involving psychopathological problems incorporate research on suicide, 51 incorporate physical problems and 35 alcohol abuse.

Of the 79 studies themed *Alcohol abuse*, only one study focused exclusively on this subject [38]. 21 studies focused primarily on alcohol abuse, with a secondary focus on other areas such as biological and genetic issues [39,40], cross-addictions [41–43], migration [44], violence [45,46], relations with parents and adults outside the family [47–50] and children’s health behaviour [51–56]. In the 57 remaining studies, alcohol use and abuse were supplemented as part of larger national public health surveys or as part of cross-national public surveys. Eighteen studies on alcohol abuse used both quantitative and qualitative approaches, 50 used only survey methods, 30 used statistical registers and 15 used qualitative methods. Out of the latter, six used a participatory approach through community and action research allocated from three research projects: the MATU project in Nuuk [30], the Paamiut Asasara project [32,57–59], and the lloqarfik Peqqissoq project in Qasigiannguit [31].

Eight studies included the theme of *Gambling abuse behaviour*, two of which as their primary focus [41,60]. Issues of gambling are related to unhealthy obsessive behaviour in all of the studies.

In the 53 studies concerning *Violence and sexual abuse*, 15 of these had this subject as their primary focus, the rest as their secondary focus to studies of risk behaviour, child neglect or prevention work. 12 studies focused primarily on violence, while seven focused primarily on sexual abuse; four of those studies concerned sexual assault on children [61–64].

In the 50 studies concerning *Suicide*, 36% had suicide as a primary focus and 63% as a secondary focus. The category includes three review studies, one with suicide as a primary focus [9] and two as a secondary focus [10,12]. Four of all the suicide themed studies used a qualitative methodology approach, one with suicide as its primary focus [65] and three as a secondary focus [34,57,66], while the remaining studies used an exclusively quantitative methodology approach.

#### Society related themes

The themes of *Postcolonial vulnerability* and *Poverty* were found in respectively 32 and 33 of the studies. The oldest studies in this review date back from 1976 [67] and 1983 [68], and the issues have been included in Greenlandic research ever since. Eight of the studies have postcolonial vulnerability [13,69–71] or poverty [63,68,72,73] as their primary focus, while the remaining use the themes as variables in their analysis and results, with other themes as their primary focus.

The 20 studies representing the theme of **Citizen and professional interaction** focused on interaction between civil citizens and different kinds of authorities. This focus in the studies is very relevant for psychology related issues, because the well-being of the locals in society is related to opportunities that often depend on the authorities. Three studies focused on interaction between school authorities and students [74–76]. Seven studies focused on interrelations between professional social workers and citizens: in these, two studies argued for additional future research and for a professional focus on children’s perspectives in matters concerning them [14,77]; one study focused on public health in the Thule region after an incident of radioactive pollution in 1968, and included as a secondary theme citizens’ mistrust in government [34]; finally, four studies covered community projects in which various groups of citizens and officials collaborated [31,32,78,79]. Eight studies focused on interaction between representatives of healthcare institutions (mostly doctors and nurses) and patients; half of these studies had the interaction as their primary focus [80–83].

93 studies included *Gender issues*, 55 of these added a definition of gender to their research as a variable in addition to other specified parameters of analysis. For example, one study on well-being among school-children [84] involved an analysis of gender-based differences in student attitudes to their own health. Gender issues continue to be an influential part of research themes in Greenland, from the oldest study in this review [85] to the most recent [86]. Within this 31 year period, nine studies were found concerning sexual health or affliction, seven concerning pregnancy and 22 concerning gender roles, family structure and violence against women.

61 studies involved *Family issues* and 56 involved *Education*. 17 studies involved both themes of education and family issues. 18 studies had family as a primary theme, and four of these used exclusively qualitative inquiry methods [87–90]. Three studies used a mix of qualitative and quantitative methods [32,36,79], seven used survey methods alone, and two were exclusively based on statistical registers [48,91]. In the theme of *Education*, 30 studies covered the attitude of the students, including analysis of their well-being and living conditions, 21 focused on the educational system and on ways of organising teaching, four focused on residential institutions [14,92–94] and one on intervention with teachers using the perspectives of their students [76]

The topic of *Resilience*, found in 26 studies, represents a relatively new focus area in Greenlandic research on children and youth, as the oldest text dates back to 2001 [30]. The research methods here were mainly qualitative, found in 18 studies, whereas an exclusively quantitative approach to survey method was found in five studies [95–99]. Four studies focused primarily on resilience in their title or stated objectives [58,78,100,101], and these were all connected to the same community project, Paamiut Asasara, conducted in Paamiut between 2008 and 2012 [32].

## Reflection upon the results

What research tells us about indigenous people is inextricably dependent upon the method of the research. Quantitative descriptive studies provide a valuable view of patterns of health behaviour and numbers of risk factors in people’s lives, society related problems, disease and suicide rates, while qualitative studies more grounded in local participation and action focus on providing knowledge about how to deal with the problems and support local resources. The results of the review analysis of the sample of 342 studies concerning children and youth in Greenland from 1976 to 2016 represent general information about the way in which research methodologies are approached and what topics are popular. Overall, there has been a development in the research material across the different approaches from an attitude of research *about* Greenland to research *in* and *for* Greenland [102]. The next upcoming tendency could be research *with* Greenland, achieved through more participation of the local citizens.

A significant majority, 72%, of studies, used quantitative research approaches in surveys, existing statistical registers or literary reviews. It is thus these kinds of methodology that for the most part represent the people of Greenland. Knowledge about children and youth in Greenland is impacted by these methodologies, and the lives of children and youth appear rather exclusively as a matter of health, whereas other aspects of life seem less visible. Only a few of the studies address the impact of participating in the public surveys or other research projects in Greenland. How do the local citizens fell about participating in survey studies? We know little about the strategies that people apply when responding to questions in interviews or written material. There are some reflections on this in a limited number of the studies, but further, substantiated reflections could be conducive.

The methodological approaches are associated with the various different scientific and theoretical research communities. Some research communities appear much more established and united, while others appear more fragmented or seemingly non-existing. Since the 1990s, medical health research in particular has been established as a united research community that represents national public health research in Greenland. This health research is usually based on methods and studies using a quantitative research approach, featuring surveys and registered statistics, albeit with increasingly qualitative approaches since 2004. Health researchers in Greenland publish attainable and coherent research results. The same large national public survey projects have been repeated (in gradually modified versions) in successive years, making it possible to discover developmental tendencies. This kind of research unity with coherent methodological approaches seems completely absent from most of the qualitative research projects. Even though a community project like Paamiut Asasara has generated 11 studies and forms a unity of coherent research, it is disconnected from other community projects in Greenland that have employed action research approaches. We assume that qualitative inquiry and community research in Greenland could benefit from a more united research approach, based on a collaborative, developmental, and sustainable research environment, for example, by focusing on building social resilience, providing knowledge about how to handle and transcend the problems and how to support local resources.

The dominance of quantitative public health science in the research concerning children and youth in Greenland produces a generalised knowledge related to sickness and health, in which details and qualitative information may be missed out. The children and youth’s own ways of formulating dreams, difficulties and accessible sides of life, as well as the way in which communities deal with these things, are seldom revealed in the quantitative public health surveys. The most prominent theme in the 342 studies was related to physical problems. And the focus on many forms of problems constituted a consistent theme throughout most of the studies. However, more recent studies seem increasingly focused on also emphasising more resourceful and positive life experiences. The new project Siunissaq uagut pigaarput (the future belongs to us) directs its focus on community strengths and components of social resilience in Tasiilaq and Nanortalik in its first annual report [103]. Focus in on building a research methodology in concurrence with local theories of knowledge. The starting point was a reflection on why the social challenges for children and young people still remain after many years of research. There seems to be a need for new methodologies to increase the results and the impact of research on children and young people in Greenland. After reviewing 341 studies that all more or less accentuate problems, it had an almost liberating effect on the authors of this mapping review to read the one study [103] that only focused on strengths and social resilience.
